# Relation of Intracerebral Hemorrhage Descriptors with Clinical Factors

**DOI:** 10.3390/brainsci10040252

**Published:** 2020-04-24

**Authors:** Kornelia M. Kliś, Roger M. Krzyżewski, Borys M. Kwinta, Krzysztof Stachura, Tadeusz J. Popiela, Jerzy Gąsowski, Jacek Długopolski

**Affiliations:** 1 Faculty of Medicine, Jagiellonian University Medical College, 31-008 Kraków, Poland; korneliakli@gmail.com; 2 Faculty of Computer Science, Electronics and Telecommunications, AGH University of Science and Technology, 30-059 Kraków, Poland; 3 TENSOR - Team of NeuroSurgery-Oriented Research, Jagiellonian University Medical College, 31-008 Kraków, Poland; jerzy.gasowski@gmail.com; 4 Department of Neurosurgery and Neurotraumatology, Jagiellonian University Medical College, 30-688 Kraków, Poland; bmkwinta@gmail.com (B.M.K.); kkstach@poczta.onet.pl (K.S.); 5 Department of Radiology, Jagiellonian University Medical College, 30-688 Kraków, Poland; msjpopie@cyf-kr.edu.pl; 6 Department of Internal Medicine and Gerontology, Jagiellonian University Medical College, 30-688 Kraków, Poland; 7 Department of Computer Science, AGH University of Science and Technology, 30-059 Kraków, Poland; dllugopol@agh.edu.pl

**Keywords:** intracerebral hemorrhage, treatment outcome, shape analysis

## Abstract

The association between intracerebral hemorrhage (ICH) shape and a poor treatment outcome has been established by few authors. We decided to analyze whether computationally assessed hemorrhage shape irregularity is associated with any known predictors of its poor treatment outcome. We retrospectively analyzed 48 patients with spontaneous intracerebral hemorrhage. For each patient we calculated Fractal Dimension, Compactness, Fourier Factor and Circle Factor. Our study showed that patients above 65 years old had significantly higher Compactness (0.70 ± 0.19 vs. 0.56 ± 0.20; *p* < 0.01), Fractal Dimension (0.46 ± 0.22 vs. 0.32 ± 0.20; *p* = 0.03) and Circle Factor (0.51 ± 0.25 vs. 0.35 ± 0.17; *p* < 0.01). Patients with hemorrhage growth had significantly higher Compactness (0.74 ± 0.23 vs. 0.58 ± 0.18; *p* < 0.01), Circle Factor (0.55 ± 0.27 vs. 0.37 ± 0.18; *p* < 0.01) and Fourier Factor (0.96 ± 0.06 vs. 0.84 ± 0.19; *p* = 0.03). In conclusion, irregularity resulting from the number of appendices can be a predictor of ICH growth; however, the size of those appendices is also important. Shape roughness better reflects the severity of brain tissue damage and a patient’s general condition.

## 1. Introduction

Spontaneous intracerebral hemorrhage (ICH) is a devastating type of stroke with a 30-day mortality rate of 30%–55% [1,2,3,4]. Therefore, it is important to establish factors that might predict a poor outcome of treatment, especially since management options for ICH remain restricted [5,6]. These predictors include poor neurological status upon admission, older age and arterial hypertension [7,8,9]. Additionally, in our previous study, which concerned the computer-aided, objective analysis of ICH shape, we found that such shape irregularity can be independently correlated with a worse treatment outcome [10]. However, mechanisms of such association are still inconclusive. One of the explanations can be found in a study by Barras et al. [11], which showed that ICH shape irregularity can be related to the risk of its enlargement, which is considered a strong predictor of treatment outcome [12]. Nonetheless, in this study, the authors used a five-grade scale based on the number of appendices with a round shape, which reflects only one of two types of tortuosity that we distinguished in our study [10]. It might also be a subjective method. As knowledge of the abovementioned predictors might also contribute to the choice of treatment method [13], we decided to analyze whether computationally assessed ICH shape irregularity can be associated with these predictors.

## 2. Materials and Methods

We performed retrospective analysis of prospectively collected data of 48 patients hospitalized between January 2013 and July 2016, with ICH confirmed by head CT scan. We included in our study only patients with spontaneous ICH, minimal or no blood in ventricles and only supra-tentorial ICH. We obtained patients’ detailed medical records, which included medical history, current medication and blood test results taken upon admission. Upon admission, patients were assessed using the Glasgow Coma Scale (GCS). The patients included in our study underwent head CT upon admission, after 24 hours and in case of neurological worsening. On discharge, they were assessed using the Glasgow Outcome Scale (GOS). We defined a poor outcome as GOS < 3. The study protocol was approved by the local bioethical committee and all patients gave their informed consent. To detect ICH contour and analyze shape irregularity we performed a series of image analyses to obtain binarized images and applied Canny Edge Detection. Then, the appropriate contour was obtained from each image. For each patient we measured four shape descriptors. The first, Compactness (C), was defined as

C = 1–(4πA/p^2^),

where *p* is the length of the perimeter of the contour and *A* is its enclosed area. It measures how efficiently a contour encloses a given area. Next, Fourier Factor (FF) was obtained from the following formula:

$$FF=1-\frac{\sum_{k=-\frac{N}{2}+1}^{N/2}\left(\frac{\left|Z_{0}\left(k\right)\right|}{\left|k\right|}\right)}{\sum_{k=-\frac{N}{2}+1}^{N/2}\left|Z_{0}\left(k\right)\right|},$$

where *k* = 0, …, N -1, *N* is the number of pixels representing the contour and *Z_0_(k)* are normalized Fourier descriptors calculated with fast Fourier transform. The result of the formula displays the irregularity of the hematoma shape as a function of the number of its Fourier series components—the greater their number is, the more irregular the ICH shape in terms of roughness. We also calculated Fractal Dimension (FD) using the Richardson method, in which FD is the slope of the linear regression model fitted to a graph of log p, where *p* is the contour perimeter, against the corresponding values of log (1/s), where s is the length of unit used to measure that perimeter. In this method, the lower the slope, the less the irregularity of the shape of hematoma. FD determines how shape contour details change according to the scale by which they are measured. The final descriptor was Circle Factor (CF), which was introduced by us. It is calculated with the following formula:

CF = p/p_c_,

where *p* is ICH contour perimeter and *p_c_* is the perimeter of the largest circle that can be inscribed into an ICH contour. According to this formula, the more the index approaches unity, the less irregular is the shape of the hematoma. All methods are described in more detail in our previous article [10]. Additionally, all the shape descriptors were normalized to lie within the 0 to 1 range. The imaging data used for analysis were obtained from the CT scans taken upon admission and prior to any surgical procedures. 

The database management and statistical analysis were performed with RStudio version 8.5 for Windows (RStudio, Inc, USA). We used the Shapiro–Wilk test to assess normality. For comparisons of continuous variables, we used the t-test for normally distributed variables and the Mann–Whitney U test for non-normally distributed variables. We used the χ2 test for dichotomized variables. To assess correlation between continuous variables, we used Pearson’s and Spearman’s correlation tests, for normally and non-normally distributed variables, respectively. To compare the predictive performance of certain shape descriptors for ICH growth, we used receiver operative characteristics (ROC) curve analysis. We express continuous variables as mean ± standard deviation. To find factors independently associated with a risk of ICH growth, we employed logistic regression analysis, with and without adjustment for possible confounders. All significance tests were two-tailed, and a 5% confidence level was considered significant. The significance was presented individually per analysis and, after a Bonferroni correction, in the context of multiple comparisons.

## 3. Results

### 3.1. Study Group Characteristics

Our study group consisted of 48 patients, 16 (33.33%) of whom were females. The average age of the study group was 63.44 ± 16.55 years. The mean GCS upon admission was 7.68 ± 4.82 and the mean GOS upon discharge was 2.42 ± 1.23. Based on CT scans, we found that the average C = 0.66 ± 0.11, the average FF = 0.87 ± 0.11, the average FD = 1.36 ± 0.13 and the average CF = 3.20 ± 0.94. A poor treatment outcome was observed in 28 (58.33%) patients.

### 3.2. Association of ICH Shape with Its Growth and Comorbidities

**Figure 1:**
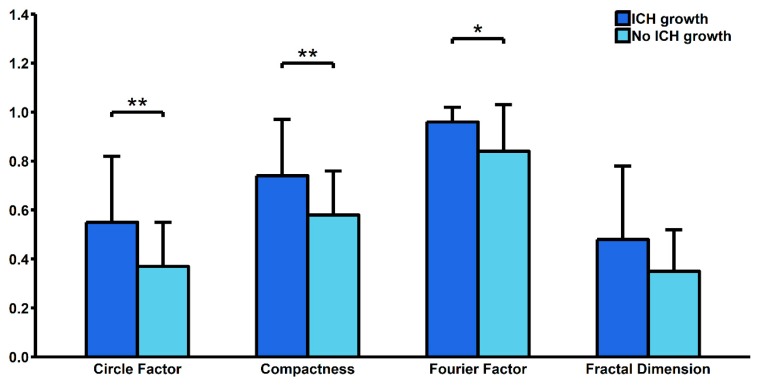
Comparison of shape descriptors between patients with and without hematoma growth. ICH—intracerebral hemorrhage. * *p* < 0.05, ** *p* < 0.01.

**Figure 2:**
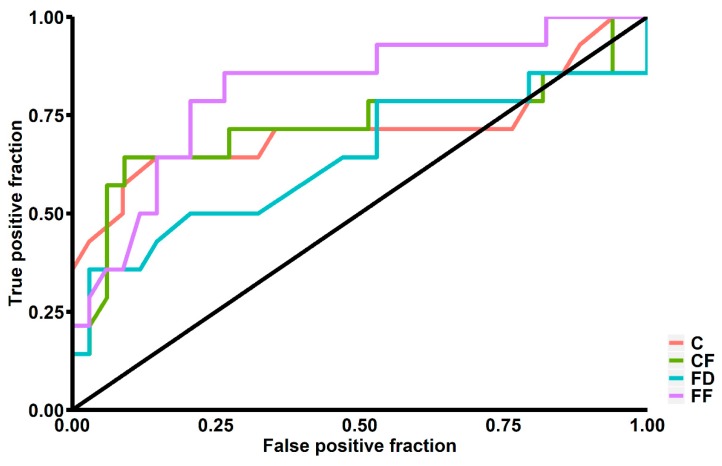
Receiver operating characteristics graph for sensitivity and specificity of intracerebral hemorrhage growth prediction. C—Compactness, CF—Circle Factor, FD—Fractal Dimension, FF—Fourier Factor.

Our study showed that patients above 65 years old had significantly higher C (0.70 ± 0.19 vs. 0.56 ± 0.20; *p* < 0.01), FD (0.46 ± 0.22 vs. 0.32 ± 0.20; *p* = 0.03) and CF (0.51 ± 0.25 vs. 0.35 ± 0.17; *p* < 0.01). We also found significantly lower FF among patients with diabetes mellitus (0.76 ± 0.34 vs. 0.89 ± 0.11; *p* = 0.049) and significantly lower CF among patients with hypercholesterolemia (0.18 ± 0.11 vs. 0.45 ± 0.22; *p* = 0.02). We found no significant differences in terms of ICH shape between patients with or without hypertension and a history of ischemic stroke (Table 1). A total of 14 (29.17%) patients had ICH growth during the first 24 hours of hospitalization. These patients had a significantly higher Mean Corpuscular Hemoglobin Concentration (34.89 ± 1.39 vs. 33.62 ± 0.95; *p* < 0.01) and a higher serum glucose level upon admission (9.97 ± 5.31 vs. 6.99 ± 2.04; *p* = 0.04). They also had significantly higher C (0.74 ± 0.23 vs. 0.58 ± 0.18; *p* < 0.01), CF (0.55 ± 0.27 vs. 0.37 ± 0.18; *p* < 0.01) and FF (0.96 ± 0.06 vs. 0.84 ± 0.19; *p* = 0.03) (Table 2, Figure 1). In multivariate logistic regression analysis, after adjustment for all possible confounders, C (OR = 2.439; 95% CI: 1.231–5.541; *p* = 0.02) and CF (OR = 1.094; 95% CI: 1.015–1.196; p = 0.03) remained independently associated with risk of ICH growth. In ROC curve analysis, we found that the area under curve (AUC) was highest for FF (AUC = 0.814), followed by CF (AUC = 0.721), C (AUC = 0.716) and FD (AUC = 0.639) (Figure 2).

### 3.3. Correlation between ICH Shape and Test Results upon Admission

**Figure 3:**
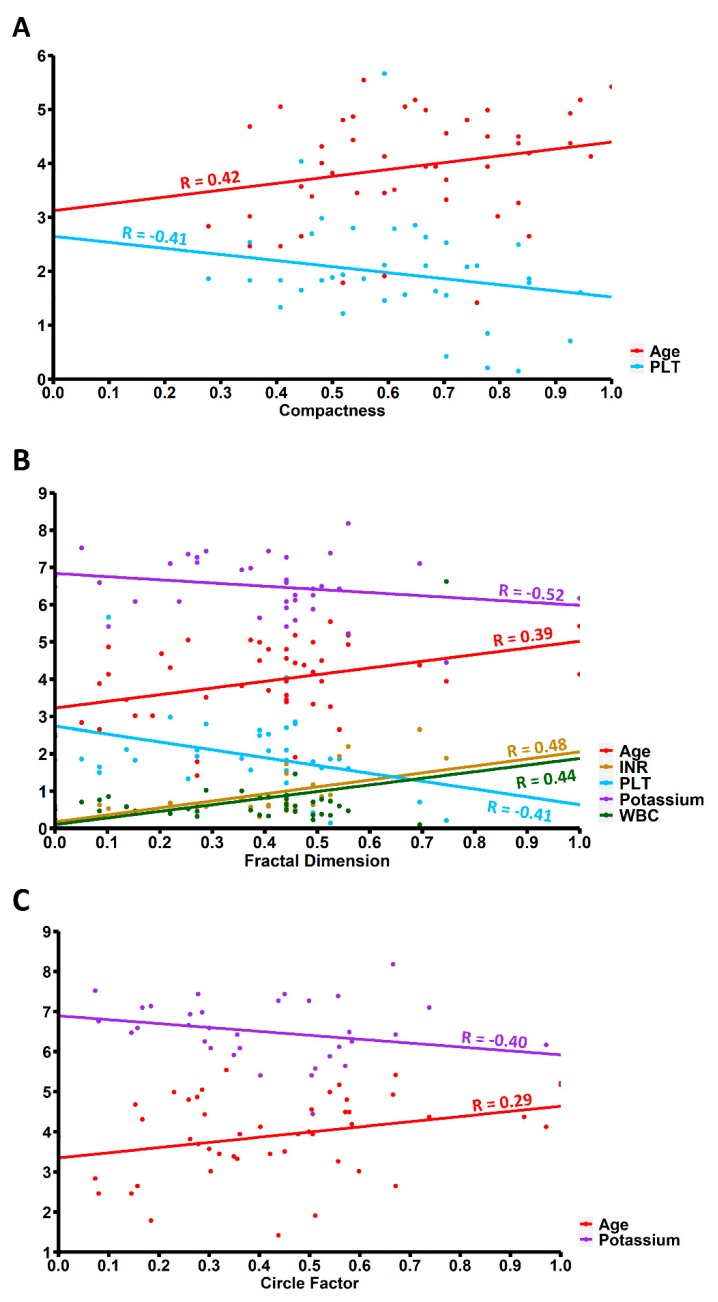
Correlation of age and blood test results upon admission with (**A**) Compactness, (**B**) Fractal Dimension and (**C**) Circle Factor. Values on x-axis are expressed as standard deviation units. WBC—White Blood Cells count, PLT—platelet count, INR—International Normalized Ratio.

In our study we found significant positive correlation between white blood cell count (WBC) upon admission and FD (R = 0.44; *p* = 0.02). We also found significant negative correlations of platelet count (PLT) upon admission with C (R = −0.41; *p* = 0.03) and FD (R = -0.38; *p* < 0.01). FD was also positively correlated with the international normalized ratio (INR) upon admission (R = 0.48; *p* < 0.01). Our study also showed significant negative correlation of potassium level upon admission with FD (R = −0.52; *p* = 0.01) and CF (R = −0.40; *p* = 0.04) (Table 3, Figure 3). Additionally, GCS upon admission was negatively correlated with FD (R = −0.37; *p* = 0.01) (Table 4).

## 4. Discussion

In our study we found associations between several risk factors of poor outcome after ICH and its shape descriptors. To analyze the ICH contour, we used computationally calculated, objective shape descriptors—three of them have previously been used in breast cancer analysis [14,15,16] and the fourth was designed by us [10]. To analyze the results of our study, it remains important to distinguish the shape characteristics defined by each of the descriptors. As mentioned before [10], there might be two main categories of shape irregularity—the first of them, which can be described by C and CF, is related to the number of appendices with a round shape, and the second, which might be described by FF and FD, is related to shape roughness. There are also differences between descriptors in both groups. As C is inversely proportional to the ICH area, it is more sensitive to the number of appendices with a regular contour than CF, which is rather sensitive to these appendices’ size. Similarly, the value of FD is the effect of contour measurements according to different scales; therefore, it is also prone to irregularities in more macroscopic shapes, as opposed to FF, which describes only the roughness of ICH margins. The CF index introduced by us has several advantages. First, it is easy to obtain, analyze and comprehend by health-care professionals without an applied math background. As compared to other indices, apart from the number of appendices as described by others, it takes into account their size, which, given the pathophysiology of peripheral bleeds, may be proportional to the severity of the extravasation of blood.

The first of our study findings was an independent correlation of C and CF with risk of ICH growth. This is consistent with the study results of Barras et al. [11]. In that study, shape irregularity was measured using a five-grade scale based on the number of appendices. As we were unable to find independent association between FF or FD, it follows that only the more macroscopic type of irregularity might be related to a risk of ICH growth. However, the association of CF also indicates that not only is the number of appendices related to ICH enlargement, but also their size. An explanation of such association might be the suggestion by Delcourt et al. that the presence of appendices is related to bleeding on hematoma borders [17].

Another finding of our study was a correlation of blood test results upon admission with ICH shape. First, we found that WBC count was positively correlated to FD. It is known that contact of blood-extravasated components with brain tissue might trigger an inflammatory response [18]. The fact that the rougher shape of ICH increases the area of brain tissue in contact with blood explains such correlation. Higher shape roughness might also be related to more severe tissue damage, which also promotes an inflammatory response [19]. Other values which were correlated with ICH shape irregularity were PLT count and INR. Clotting disorders might cause more severe bleeding and therefore a higher pressure of ICH formation, which could also lead to a rougher shape of hematoma. That explanation might be consistent with the finding that both INR and PLT were correlated with FD. Additionally, the abovementioned fact that bleeding at the hematoma border might be related to the number of appendices explains the correlation between C and PLT count. In terms of potassium, it was shown that hypokalemia is a common phenomenon among patients with brain damage [20]. The mechanisms of a low level of potassium after brain injury remain unclear; however, it was suggested that it might result from electrolytes shifting from an extracellular to an intracellular compartment, stimulated by the release of catecholamines [20]. Therefore, potassium level might as well reflect severity of brain damage.

Our study also showed an association between older age and C, CF and FD. It is already known that changes in cerebral vasculature that occur with age make cerebral tissue more susceptible to injury [21]. Vascular walls weakening might also contribute to more severe bleeding and therefore irregularity of shape expressed both in roughness and presence of appendices.

Another interesting finding of our study was a negative correlation between GCS upon admission and FD. From all the shape descriptors, FD was shown to be related to the largest number of possible indicators of brain tissue damage and bleeding severity, which explains its association with patients’ condition. As mentioned before, that descriptor most comprehensively describes the irregularity of ICH shape. A similar approach to shape analysis was introduced in a study by Wang et al. [22], where shapes were classified either as regular or irregular based on both appendices and roughness. Those studies found a correlation of ICH shape irregularity with 30-days mortality. However, in that kind of classification, it might be difficult to find clear borders between regular and irregular shape. Additionally, in our previous study, FD was shown to be independently related to a higher risk of poor treatment outcome.

## 5. Conclusions

Computer-aided analysis of ICH allows researchers to objectively analyze its shape irregularity in a few different contexts. As previously shown, irregularity resulting from the number of appendices can be a predictor of ICH growth; however, the size of those appendices is also important. Shape roughness better reflects the severity of brain tissue damage and a patient’s general condition. Therefore, a comprehensive descriptor of shape such as FD remains best for that kind of analysis. On the other hand, FF, which takes into consideration only roughness without considering entire shape, was not correlated with any of the analyzed factors. Our study was mainly limited by the study group size. Further research should be performed on a bigger and more varied study group. Despite that limitation, this is the first study that analyzes the association between objective shape descriptors and potential predictors of poor treatment outcome after ICH.

## Figures and Tables

**Table 1:** Association of comorbidities with ICH shape descriptors. SD—standard deviation.

	**Age > 65 years** **(*n* = 23)**	**Age < 65 years** **(*n* = 25)**	***p*-Value**
Compactness ± SD	0.70 ± 0.19	0.56 ± 0.20	< 0.01^1^
Fourier Factor ± SD	0.89 ± 0.11	0.86 ± 0.21	0.45^1^
Fractal Dimension ± SD	0.46 ± 0.22	0.32 ± 0.20	0.03^1^
Circle Factor ± SD	0.51 ± 0.25	0.35 ± 0.17	< 0.01^1^
	**Female gender** **(*n* = 16)**	**Male gender** **(*n* = 32)**	***p*-Value**
Compactness ± SD	0.63 ± 0.16	0.63 ± 0.23	0.99^1^
Fourier Factor ± SD	0.86 ± 0.12	0.88 ± 0.19	0.74^1^
Fractal Dimension ± SD	0.41 ± 0.18	0.38 ± 0.24	0.67^1^
Circle Factor ± SD	0.41 ± 0.23	0.43 ± 0.23	0.76^1^
	**Hypertension** **(*n* = 18)**	**No hypertension** **(*n* = 30)**	***p*-Value**
Compactness ± SD	0.61 ± 0.26	0.64 ± 0.16	0.58^1^
Fourier Factor ± SD	0.86 ± 0.25	0.88 ± 0.10	0.65^1^
Fractal Dimension ± SD	0.39 ± 0.24	0.39 ± 0.21	0.97^1^
Circle Factor ± SD	0.42 ± 0.26	0.43 ± 0.21	0.96^1^
	**Diabetes mellitus** **(*n* = 7)**	**No diabetes mellitus** **(*n* = 41)**	***p*-Value**
Compactness ± SD	0.57 ± 0.35	0.64 ± 0.17	0.43^1^
Fourier Factor ± SD	0.76 ± 0.34	0.89 ± 0.11	0.05^1^
Fractal Dimension ± SD	0.42 ± 0.32	0.38 ± 0.20	0.63^1^
Circle Factor ± SD	0.40 ± 0.34	0.43 ± 0.21	0.72^1^
	**Hypercholesterolemia** **(*n* = 4)**	**No hypercholesterolemia** **(*n* = 44)**	***p*-Value**
Compactness ± SD	0.42 ± 0.13	0.65 ± 0.2	0.03^1^
Fourier Factor ± SD	0.83 ± 0.14	0.88 ± 0.17	0.56^1^
Fractal Dimension ± SD	0.25 ± 0.20	0.40 ± 0.22	0.20^1^
Circle Factor ± SD	0.18 ± 0.11	0.45 ± 0.22	0.02^1^
	**History of ischemic stroke** **(*n* = 3)**	**No history of ischemic stroke** **(*n* = 45)**	***p*-Value**
Compactness ± SD	0.56 ± 0.18	0.63 ± 0.21	0.53^1^
Fractal Dimension ± SD	0.89 ± 0.04	0.87 ± 0.17	0.89^1^
Circle Factor ± SD	0.36 ± 0.15	0.39 ± 0.22	0.84^1^
Compactness ± SD	0.31 ± 0.16	0.43 ± 0.23	0.35^1^

^1^Bonferroni adjusted 5% significance level was 0.05/24 = 0.0021.

**Table 2:** Comparison of patients with and without ICH growth. SD—Standard Deviation.

Risk factor	ICH Growth (*n* = 14)	No ICH Growth (*n* = 34)	*p*-Value
Age [years] ± SD	66.71 ± 15.47	62.39 ± 16.61	0.41^1^
Female gender [%]	21.43 (3)	38.24 (13)	0.26^1^
**Comorbidities**			
Hypertension [%]	50 (7)	32.35 (11)	0.25^1^
Diabetes mellitus [%]	14.29 (2)	14.71 (5)	0.97^1^
Alcoholism [%]	0 (0)	2.94 (1)	0.52^1^
Ischemic heart disease [%]	7.14 (1)	11.76 (4)	0.63^1^
History of heart attack [%]	0 (0)	5.88 (2)	0.35^1^
History of ischemic stroke [%]	0 (0)	8.82 (3)	0.25^1^
Atrial fibrillation [%]	14.29 (2)	11.76 (4)	0.81^1^
Lungs diseases [%]	0 (0)	2.94 (1)	0.52^1^
Hyperthyroidism [%]	7.14 (1)	2.94 (1)	0.51^1^
Hypothyroidism [%]	14.29 (2)	5.88 (2)	0.34^1^
Hypercholesterolemia [%]	0 (0)	11.76 (4)	0.18^1^
**Current medications**			
Acetylsalicylic acid [%]	14.29 (2)	11.76 (4)	0.81^1^
Β—blockers [%]	14.29 (2)	14.71 (5)	0.97^1^
Angiotensin-converting-enzyme inhibitors [%]	7.14 (1)	8.82 (3)	0.85^1^
Calcium channel blockers [%]	0 (0)	8.82 (3)	0.25^1^
Diuretics [%]	14.29 (2)	29.41 (10)	0.27^1^
Steroids [%]	0 (0)	5.88 (2)	0.35^1^
Antidiabetic therapy [%]	7.14 (1)	0 (0)	0.12^1^
Insulin [%]	0 (0)	2.94 (1)	0.52^1^
Anticoagulants [%]	28.57 (4)	20.59 (7)	0.55^1^
Statins [%]	0 (0)	11.76 (4)	0.18^1^
**Hematoma measurements**			
Compactness ± SD	0.74 ± 0.23	0.58 ± 0.18	<0.01
Fourier Factor ± SD	0.96 ± 0.06	0.84 ± 0.19	0.03
Fractal Dimension ± SD	0.48 ± 0.3	0.35 ± 0.17	0.07
Circle Factor ± SD	0.55 ± 0.27	0.37 ± 0.18	<0.01^1^
Volume - ABC/2 [mm3] ± SD	32086.46 ± 31745.84	56904.27 ± 40666.45	0.19^1^
Precise volume [mm3] ± SD	34942 ± 7635.34	62833.16 ± 37010.62	0.35^1^
**Blood tests results upon admission**			
White Blood Cells count [103/μL] ± SD	9.92 ± 4.8	14.61 ± 20.39	0.48^1^
Red Blood Cells count [103/μL] ± SD	4.23 ± 1.07	4.25 ± 0.72	0.95^1^
Platelet count [103/μL] ± SD	179.4 ± 67.4	189.11 ± 103.54	0.78^1^
Haemoglobin [g/dl] ± SD	13.16 ± 3.28	12.67 ± 2.16	0.60^1^
Mean Corpuscular Volume [μm3] ± SD	89.33 ± 4.86	88.8 ± 5.63	0.79^1^
Mean Corpuscular Haemoglobin [pg] ± SD	31.15 ± 2.04	29.88 ± 2.05	0.10^1^
Mean Corpuscular Haemoglobin Concentration [g/dL] ± SD	34.89 ± 1.39	33.62 ± 0.95	<0.01^1^
International Normalized Ratio ± SD	12.56 ± 35.64	1.76 ± 2.12	0.14^1^
Prothrombin Time [s] ± SD	17.4 ± 8.95	14.88 ± 6.55	0.36^1^
Activated Partial Thromboplastin Time [s] ± SD	36.13 ± 10.84	32.57 ± 9.63	0.35^1^
Sodium [mmol/L] ± SD	138.4 ± 4.62	141.07 ± 6.63	0.25^1^
Potassium [mmol/L] ± SD	3.84 ± 0.48	3.97 ± 0.63	0.55^1^
Glucose [mmol/L] ± SD	9.97 ± 5.31	6.99 ± 2.04	0.04^1^
Creatinine [μmol/L] ± SD	71.58 ± 17.94	93.63 ± 72.69	0.31^1^
Urea [mmol/L] ± SD	4.95 ± 1.96	8.04 ± 6.18	0.12^1^

^1^Bonferroni adjusted 5% significance level was 0.05/44=0.0011.

**Table 3:** Correlation of ICH shape with blood test results taken upon admission.

	Compactness	Fourier Factor	Fractal Dimension	Circle Factor
White Blood Cells count [103/μL]	0.17	−0.16	0.44	0.15
*p*-value^1^	0.40	0.41	0.02	0.46
Red Blood Cells count [103/μL]	0.02	0.21	−0.05	0.01
*p*-value^1^	0.94	0.29	0.82	0.97
Haemoglobin [g/dL]	0.02	0.23	−0.10	0.01
*p*-value^1^	0.93	0.26	0.63	0.99
Platelet count [103/μL]	−0.41	−0.01	−0.38	−0.30
*p*-value^1^	0.03	0.96	0.049	0.13
International Normalized Ratio	0.29	0.14	0.48	0.22
*p*-value^1^	0.09	0.44	< 0.01	0.20
Activated Partial Thromboplastin Time [s]	−0.11	−0.19	−0.10	0.07
*p*-value^1^	0.58	0.34	0.61	0.73
Sodium [mmol/L]	0.15	−0.02	0.26	0.11
*p*-value^1^	0.46	0.91	0.20	0.59
Potassium [mmol/L]	−0.29	0.04	-0.52	−0.40
*p*-value^1^	0.14	0.83	0.01	0.04

^1^Bonferroni adjusted 5% significance level was 0.05/32 = 0.0016.

**Table 4:** Factors correlated with the Glasgow Coma Scale Score upon admission.

	R	*p*-Value
Hematoma volume [mm3]	0.28	0.47
Age	−0.21	0.59
Compactness	−0.18	0.24
Fourier Factor	0.02	0.91
Fractal Dimension	−0.37	0.01
Circle Factor	−0.19	0.20
